# A Picture on the Wall: Innovative Mapping Reveals Cold-Water Coral Refuge in Submarine Canyon

**DOI:** 10.1371/journal.pone.0028755

**Published:** 2011-12-14

**Authors:** Veerle A. I. Huvenne, Paul A. Tyler, Doug G. Masson, Elizabeth H. Fisher, Chris Hauton, Veit Hühnerbach, Timothy P. Le Bas, George A. Wolff

**Affiliations:** 1 Marine Geoscience, National Oceanography Centre, Southampton, United Kingdom; 2 School of Ocean and Earth Sciences, University of Southampton, Southampton, United Kingdom; 3 School of Environmental Sciences, University of Liverpool, Liverpool, United Kingdom; Heriot-Watt University, United Kingdom

## Abstract

Cold-water corals are azooxanthellate species found throughout the ocean at water depths down to 5000 m. They occur in patches, reefs or large mound structures up to 380 m high, and as ecosystem engineers create important habitats for a diverse fauna. However, the majority of these habitats are now within reach of deep-sea bottom trawling. Many have been severely damaged or are under threat, despite recent protection initiatives. Here we present a cold-water coral habitat type that so far has been overlooked – quite literally – and that has received minimal impact from human activities. Vertical and overhanging cliffs in deep-sea canyons, revealed using an innovative approach to marine habitat mapping, are shown to provide the perfect substratum for extensive cold-water coral-based communities. Typical canyon-related processes, including locally enhanced internal tides and focussed downslope organic carbon transport, provide favourable environmental conditions (current regime, food input) to sustain the communities, even outside the optimal depth and density envelopes reported elsewhere in the NE Atlantic. Our findings show that deep-sea canyons can form natural refuges for faunal communities sensitive to anthropogenic disturbance, and have the potential to fulfil the crucial role of larval sources for the recolonisation of damaged sites elsewhere on the margin.

## Introduction

Contrary to common perception, more coral species have been described from the deep sea than from shallow tropical waters [1]. Cold-water corals (CWCs) are azooxanthellate filter-feeders, i.e. lacking symbiotic algae, from the anthozoan orders Scleractinia (stony corals), Octocorallia (soft corals), Anthipatharia (black corals) and the hydrozoan family Stylasteridae (hydrocorals) [2]. Colonial and solitary species are found on continental margins, seamounts and mid-ocean ridges, in water depths from a few metres to >5000 m [3]. Although several solitary species can live in muddy environments, most species need a hard substratum for settlement and locally enhanced currents to ensure sufficient food input. The most common scleractinian coral in the NE Atlantic, *Lophelia pertusa*, occurs in waters of 4 to 12°C, in the potential density (σ_θ_) envelope 27.35–27.65 kgm^−3^, and in areas where oceanographic processes such as internal waves focus increased surface primary production to specific seabed locations [4], [5]. The significance of CWCs is their ability to form structural habitats including patches, reefs or carbonate mounds up to 380 m high [6], [7]. However, with commercial deep-sea trawling now frequently reaching depths of 1500 m, many reefs have been damaged or destroyed [8]. Reef destruction not only reduces alpha (local) diversity but also has an impact on sexual reproduction in corals [9]. Occasionally, reported damage has triggered conservation measures (e.g. the Darwin Mounds offshore Scotland [10], or reefs offshore Norway [11]). However, it is unsure how well these reefs will recover, while many others, especially those in international waters, are still unprotected.

**Figure 1:**
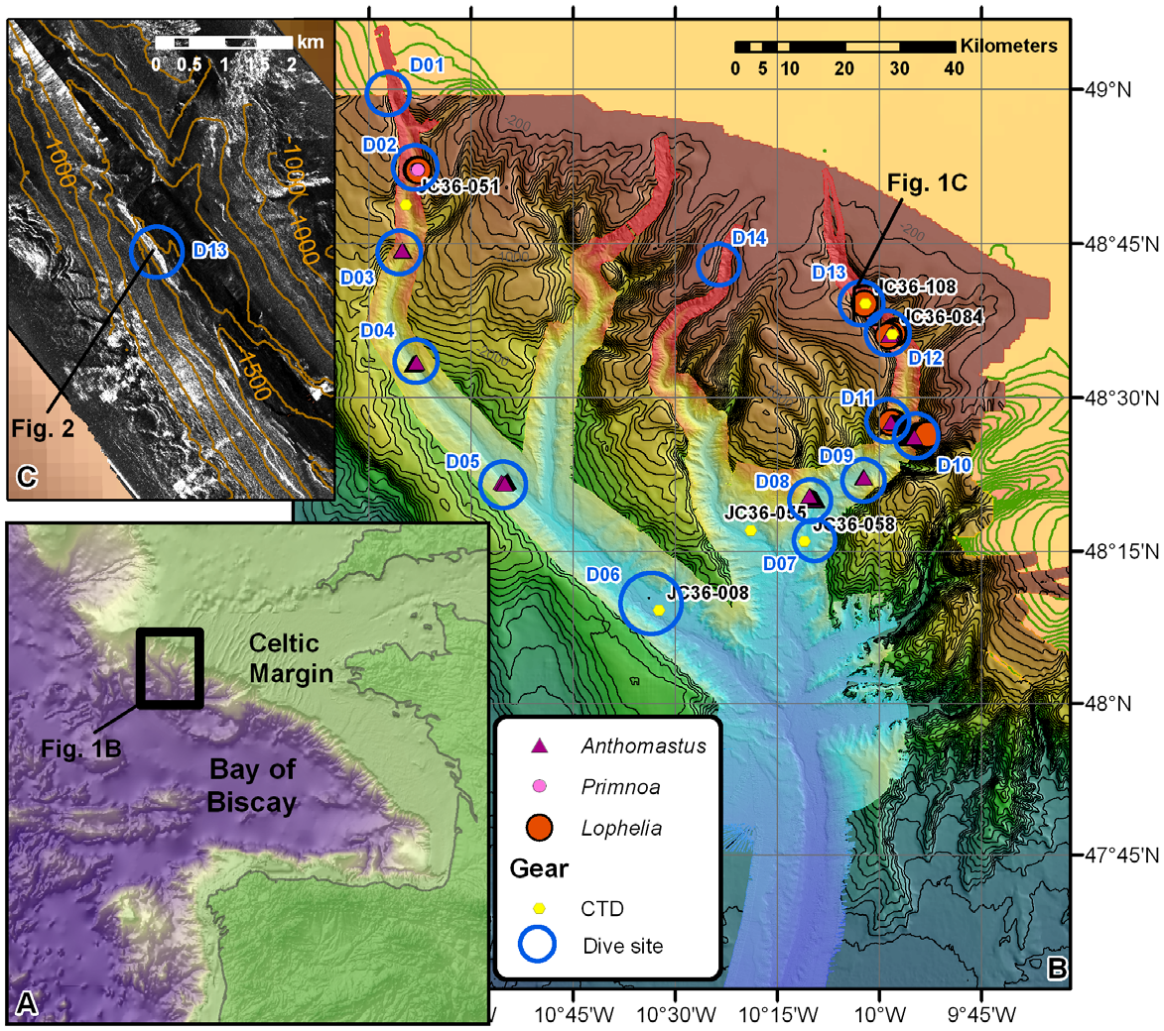
Overview map of Whittard Canyon and the main study areas. **A** Location map of Whittard Canyon on the Celtic Margin, Bay of Biscay. **B** Bathymetric map of Whittard Canyon, with indication of sampling stations. Background bathymetry from GEBCO [36] (pastel colours, 1.2×1.8 km pixel size) and from the Irish National Seabed Survey (darker colours, 200 m pixels, kindly provided by the Geological Survey of Ireland, Dublin). Contour interval 100 m. Detailed bathymetry, collected during our surveys, in foreground (bright colours, 50 m pixels). Blue circles indicate dive sites, labelled D**; CTD stations are labelled JC36-***. Occurrences of live specimens of the main coral species are marked. **C** Detail of 30 kHz TOBI sidescan sonar imagery at dive site D13, illustrating the occurrence of steep cliffs along the canyon walls (elongated high backscatter patches, represented in light colours). Bathymetric contours at 100 m intervals.

The continental margin of the Bay of Biscay is incised by a large number of submarine canyons (Fig. 1), and has been identified as a suitable and potentially important habitat for CWC [12]. Canyons are the main sediment transport pathways between the shelf and the deep sea. Processes including the capture of along-shelf sediment transport, resuspension by internal waves and tides [13], dense shelf water cascading (DSWC [14]) and turbidity currents may result in either canyon flushing or focussed deposition of sediments and organic matter [15]. Canyons are complex environments that can harbour a significantly increased biodiversity and biomass compared to the open slope [16], [17]. However, this terrain heterogeneity also makes them challenging environments for study. Conventional shipboard mapping and sampling techniques cope poorly with the steep topography. In the Bay of Biscay, this has caused difficulties in the assessment of the present-day status of CWCs compared to historical records [12]. The increasing availability of remotely operated vehicles (ROVs) and autonomous underwater vehicles (AUVs) now provides the opportunity to fill this knowledge gap [18].

An extensive ROV-based habitat mapping programme was carried out in Whittard Canyon in the northern Bay of Biscay, between 500 and 4000 m water depth (mwd) (Fig. 1, see method section). The main aim was to identify the different habitats in the canyon, with emphasis on CWC communities, and to assess their status, especially with respect to human impacts. Whittard Canyon is a dendritic system, connecting the broad shelf at ca. 200 mwd with the Whittard Channel and Celtic Fan at 4000 mwd [19]. Canyon incision, mainly by headward erosion and retrogressive slope failure, started in the Plio-Pleistocene and cut deeply into the underlying Miocene deltaic deposits and Cretaceous/Paleocene chalks. The developing canyon morphology was influenced by the location of existing NNW-SSE trending fault systems, older buried canyons, and natural depressions in the seafloor [20]. The canyon was very active during sealevel lowstands, more particularly during deglaciation phases [19], [21]. However, its present-day activity is much reduced: it is located far from terrestrial sources, and sediment input is limited to shelf spill-over [20]. Our mapping programme in the canyon resulted in the successful identification of several CWC habitats, including settings of a type and extent not yet reported to science before.

## Results and Discussion

CWCs were found in water depths ranging from 880 to 3300 m. They were dominated by the soft coral *Anthomastus* sp, the scleractinian coral *Lophelia pertusa* and the octocorals *Primnoa* sp., *Acanthogorgia* sp. and *Acanella* sp. (Fig. 1). Most corals appear to occur on locally steeper slopes (e.g. cliffs, ledges, or large boulders), although some patches were also found on relatively level surfaces. *L. pertusa* was found at 5 sites, between 1300 and 1880 mwd. The highest *Lophelia* density, and visually most diverse community, was encountered in a new type of setting at ca. 1350 mwd in the eastern branch of the canyon, on a 1600 m long cliff identified on the shipboard multibeam and sidescan sonar maps (Fig. 1c). *Lophelia pertusa* has been reported from steep canyon sites elsewhere, including the Bay of Biscay [18] and the NW Mediterranean [22], but in those cases was limited to patchy occurrences. No communities of similar density and extent, and from similar cliff settings as found here, have been described so far. To investigate the spatial characteristics of this new type of coral habitat, morphological maps of the cliff were produced at different levels of resolution, using a newly developed approach in which the ROV-mounted multibeam was installed in a forward-looking, rather than the normal downward-looking, configuration (see methods section for details).

**Figure 2:**
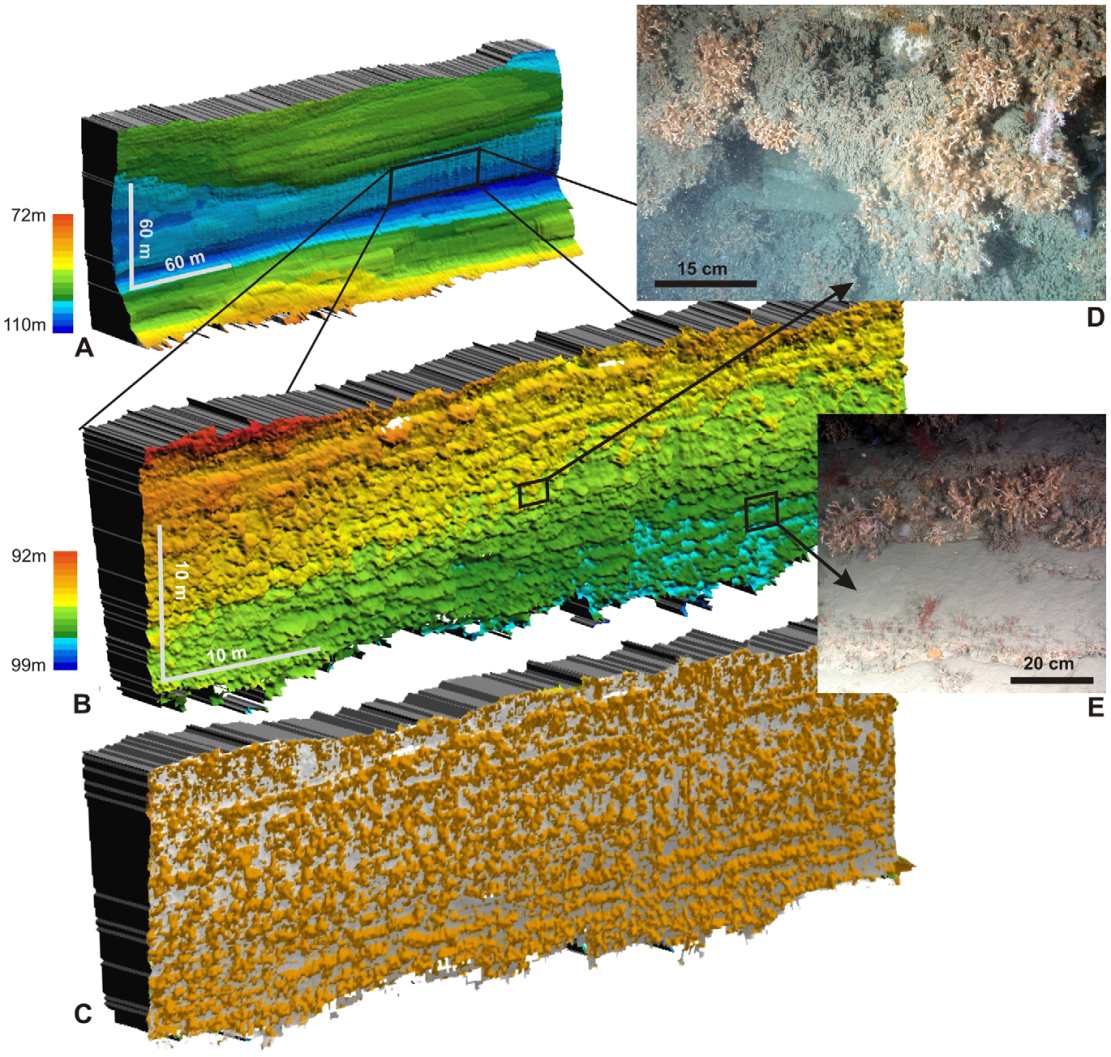
Morphology and photographs of the main cold-water coral community in Whittard Canyon. **A** Cliff morphology at dive site D13 mapped from 30 m distance using the forward-looking multibeam configuration on the ISIS ROV. Pixel size 0.5 m. Colour scale indicates distance from arbitrary vertical plane roughly parallel to the cliff and ROV track. **B** Cliff morphology mapped at 7 m distance, showing individual coral colonies. Sub-horizontal lineations correspond to alignment of coral colonies on individual protruding rock strata. Pixel size 0.1 m. **C** Top-hat transformation of previous map, indicating coral coverage of ∼70% shaded in orange. **D.** Representative photograph showing large *Lophelia* colonies up to 0.5 m diameter, plus typical associated fauna. **E** Illustration of coral preference for specific strata and ledges.

The overview maps show a 120 m high cliff, overhanging by ∼20 m (Fig. 2a). Individual strata are resolved in the higher-resolution images. On the most detailed map, individual coral colonies can be identified (Fig. 2b, c) and faunal coverage can be estimated at ∼70%. Hence this coral community is of similar density and extent (∼0.192 km^2^) as the live coral cover on Thérèse Mound (∼0.25 km^2^), one of the richest CWC mounds in the Porcupine Seabight, W of Ireland [23], [24]. It is clear that the 3D morphology of overhangs cannot be mapped correctly with ship-borne acoustic instruments. Even steep cliffs or stepped morphologies are generally not represented accurately, because of the limited spatial resolution of acoustic systems in deep water and the associated smoothing effect of the gridding process. Based on this understanding, we estimate that all areas sloping >35° on the ship-borne bathymetry map could potentially contain sites for near-vertical, *Lophelia*-based communities. Such slopes cover 9.4 km^2^, or ∼1.35% of the 3D surface area within the depth range of *Lophelia* in Whittard Canyon (<2000 mwd). Again, this is in the same order of magnitude as the present-day mound surfaces in the Belgica CWC Mound province in the Porcupine Seabight (∼12 km^2^, or 1.95% of the province area) [24]. Considering the large number of submarine canyons in the Bay of Biscay (Fig. 1a), these findings suggest that vertical coral reefs could form a significant contribution to the CWC reef occurrence in the NE Atlantic.

**Figure 3:**
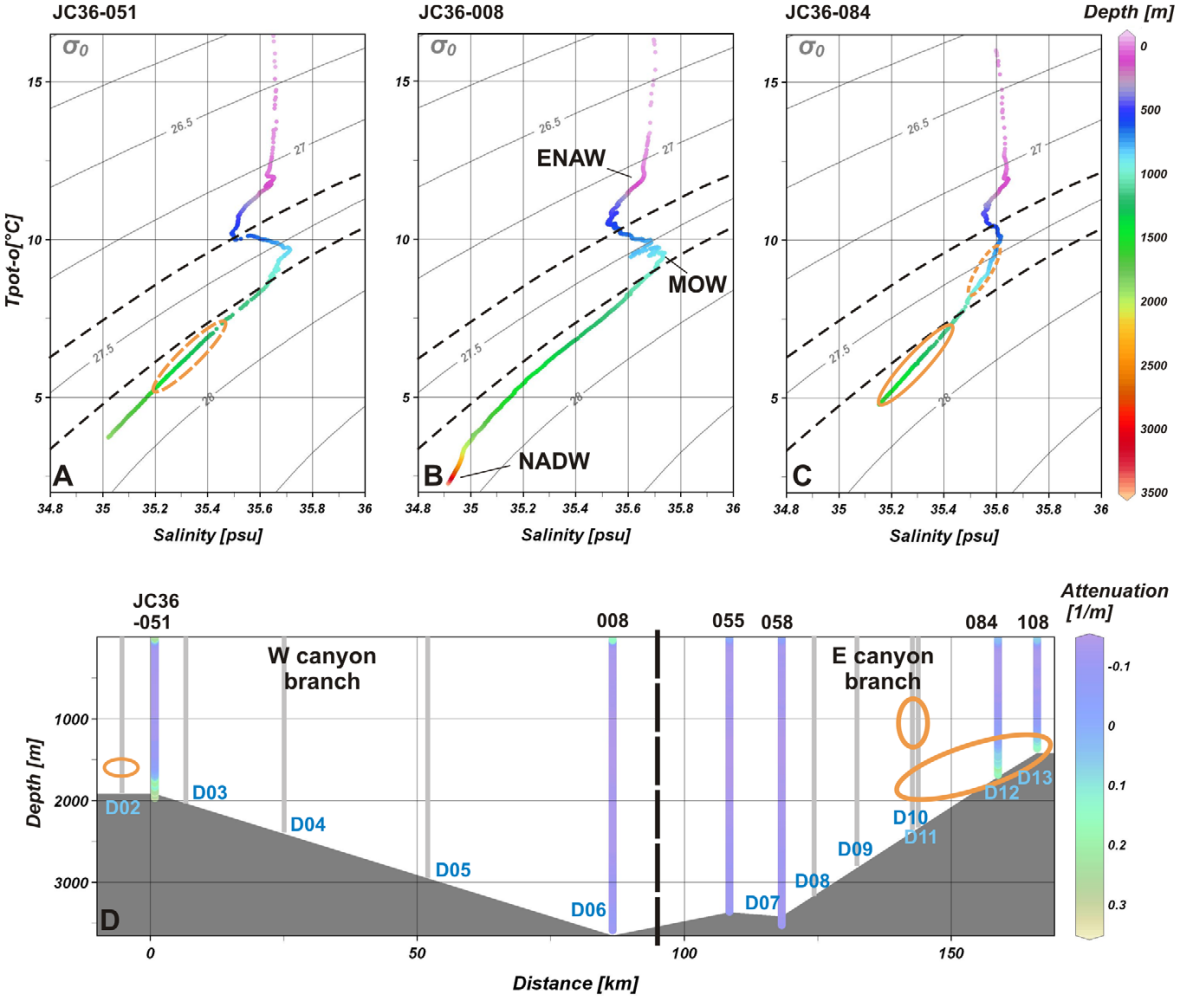
Water mass properties in Whittard Canyon. **A–C** Potential temperature – salinity graphs of CTD stations JC36-051, 008 and 084. Colour scale indicates depth, isopycnals show the potential density structure (referred to 0 dbar). Heavy dashed lines delineate the optimal potential density envelope for *Lophelia pertusa* in the NE Atlantic according to Dullo et al. [4]. Main water masses: ENAW: Eastern North Atlantic Water; MOW: Mediterranean Outflow Water, NACW: North Atlantic Central Water [37]. *Lophelia* occurrences are indicated by orange ellipses (broken line: projected from a nearby station). Note that all recorded *Lophelia* occurrences are located below the salinity maximum of the MOW, which appears shallower and less well expressed in the eastern branch than in the rest of the canyon, and that all but one of the sites occur below the optimal potential density envelope. **D** Light attenuation profiles through the western and eastern branch of Whittard Canyon, showing turbidity maxima in the upper/middle canyon. Dive sites without CTD information indicated with grey bars, coral occurrences indicated by orange ellipses.

The necessary food input to sustain such an extensive CWC community derives from the oceanographic characteristics of the area and the focussing effect of the canyon. The Celtic Margin is known as a region of enhanced primary production during the spring bloom, and part of this production is exported to deeper waters [25]. Canyons can act as sediment and organic matter traps [15], and the Whittard Canyon floor is locally enriched in particulate organic carbon and phytodetritus (chlorophyll a) in comparison to the open slope [26]. Bottom nepheloid layers are found in the middle and upper canyon branches (1200–2000 mwd, Fig. 3d). The suspended particulate organic matter (sPOM) in those layers, close to the main coral communities, has high levels of particulate organic carbon (POC; Table 1), well within the optimum range for CWC in the NE Atlantic (∼0.8–4 µM) [27]. The sPOM is also lipid-rich, and the high proportion of labile lipids suggests a high food quality. In the eastern branch, the lipids include the essential fatty acids docosahexaenoic acid (DHA) and eicosapentaenoic acid (EPA), probably derived from phytoplankton or zooplankton detritus [28], [29]. In the western branch, the sPOM contains a high proportion of monounsaturated fatty acids, e.g. 9(Z)-octadecenoic acid. Samples collected further down the canyon, where *Lophelia* is absent, have lower POC and total lipid concentrations (Table 1). The lipids here are dominated by semi-labile and refractory compounds (saturated fatty acids, sterols and *n*-alkanols), suggesting a greater proportion of reworked/resuspended sPOM in this area. This is substantiated by low molar C/N ratios (4.1–5.2), which are typical for oxidized deep-sea sediments [30]. Although these samples only provide “snapshots” of the sPOM in the canyon, our findings are further corroborated by a relatively higher proportion of labile lipids such as EPA, DHA and 9(Z)-octadecenoic acid in replicate surficial sediment samples collected at coral sites, compared to most sites devoid of corals (except for site D07; Table 2).

**Table 1:** Organic matter characterisation of the bottom waters of Whittard Canyon.

CTD/SAP station	Sample depth (mwd)	POC (µM)	Total Lipid concentration µgm^−3^ (µg gTOC^−1^)	Molar C/N ratios	EPA µgm^−3^ (µg gTOC^−1^)	DHA µgm^−3^ (µg gTOC^−1^)	C_18∶1_ Z-D^9^ µg m^−3^ (µg gTOC^−1^)
JC36-008	3586	0.5	35.4 (6620.7)	5.2	0.6 (115)	0.3 (47.3)	4.4 (814)
JC36-051*	1953	1.9	228.3 (9931.9)	7.7	4.4 (190.8)	2.3 (100.2)	139.4 (6064.3)
JC36-055	3347	0.3	22.3 (6766.9)	4.6	0.8 (239.0)	0.9 (286.1)	2.0 (609.5)
JC36-058	3507	0.3	46.0 (1488.2)	4.1	0.8 (272.3)	1.9 (609.2)	3.5 (1136.7)
JC36-084*	1702	1.0	618.8 (52445.4)	6.1	61.3 (5196.4)	99.8 (8456.7)	51.0 (4318.7)

Suspended organic matter in the water column, within ∼10 m of the canyon floor, was sampled with a Stand-Alone Pump (SAP), deployed at the CTD stations. POC: particulate organic carbon; TOC: total organic carbon;

*stations/sites in the vicinity of cold-water coral communities.

**Table 2:** Organic matter characterisation of seabed sediments in Whittard Canyon.

ROV Dive Site	Sample depth (mwd)	TOC (mmolg^−1^)	Total Lipid concentration ngg^−1^ (µg gTOC^−1^)	Molar C/N ratios	EPA ng g^−1^ (µg gTOC^−1^)	DHA ng g^−1^ (µg gTOC^−1^)	C_18∶1_ Z-D^9^ ng g^−1^ (µg gTOC^−1^)
D01	1065	0.1	49032.3 (41648.3)	13.0	0.0 (0.0)	0.0 (0.0)	24.9 (21.1)
D02*	1385	0.7	37961.7 (4740.6)	9.7	690.4 (87.4)	561.6 (73.3)	1042.2 (131.9)
D05*	3296	0.6	22386.5 (3043.9)	9.5	163.9 (22.3)	0 .0 (0.0)	2377.8 (323.3)
D06	3646	0.6	14660.4 (2375.9)	8.3	54.6 (10.0)	22.9 (4.7)	193.2 (31.9)
D07	3411	0.6	25792.0 (2820.5)	9.5	1275.1 (140.3)	559.0 (61.3)	265.8 (28.9)
D11*	2483	0.2	11541.1 (4265.5)	7.6	431.7 (161.5)	200.8 (74.6)	239.8 (88.7)
D12*	1299	0.7	29870.2 (4409.7)	8.6	1747.9 (307.2)	1095.8 (166.3)	634.3 (105)

Sediment samples were obtained from ROV pushcores. Data presented are for the surficial sediments only (0–0.5 cm). POC: particulate organic carbon; TOC: total organic carbon;

*stations/sites in the vicinity of cold-water coral communities.

The coral cliff habitats exhibit some fundamental differences compared to the better-known mounds, reefs and patches [3]. Within the canyon, only one of the *Lophelia* sites occurs in the optimal potential density envelope as described by Dullo et al. [4] for the NE Atlantic, while the richest CWC communities are found in deeper and denser waters (27.74–27.84 kg m^−3^, Fig. 3a–c). We propose that these settings, so far unique for the NE Atlantic margin, result from processes that typify canyons. Although the exact mechanism behind the apparent coincidence between *Lophelia* occurrence and water column characteristics is not yet clear, it has been suggested that the optimal density envelope, which generally corresponds to a sharp pycnocline [5], may be instrumental in concentrating food particles, or may be important for the lateral transport of *Lophelia* larvae [4]. It appears that in canyons, downslope transport processes such as dilute gravity currents, DSWC or internal tides can transport both food particles and larvae beyond that boundary, to greater depths where the canyon still provides the required hard substrata, enhanced particle concentrations and enhanced (tidal) currents. It has to be noted that *Lophelia* colonies have also been described from the Gulf of Mexico, thriving in environmental conditions outside the potential density envelope which appears to characterise the NE Atlantic [31]. This observation, together with our finding in the Whittard Canyon, underlines once more that a better understanding of the relationship between CWC growth and physical oceanography is urgently needed.

**Figure 4:**
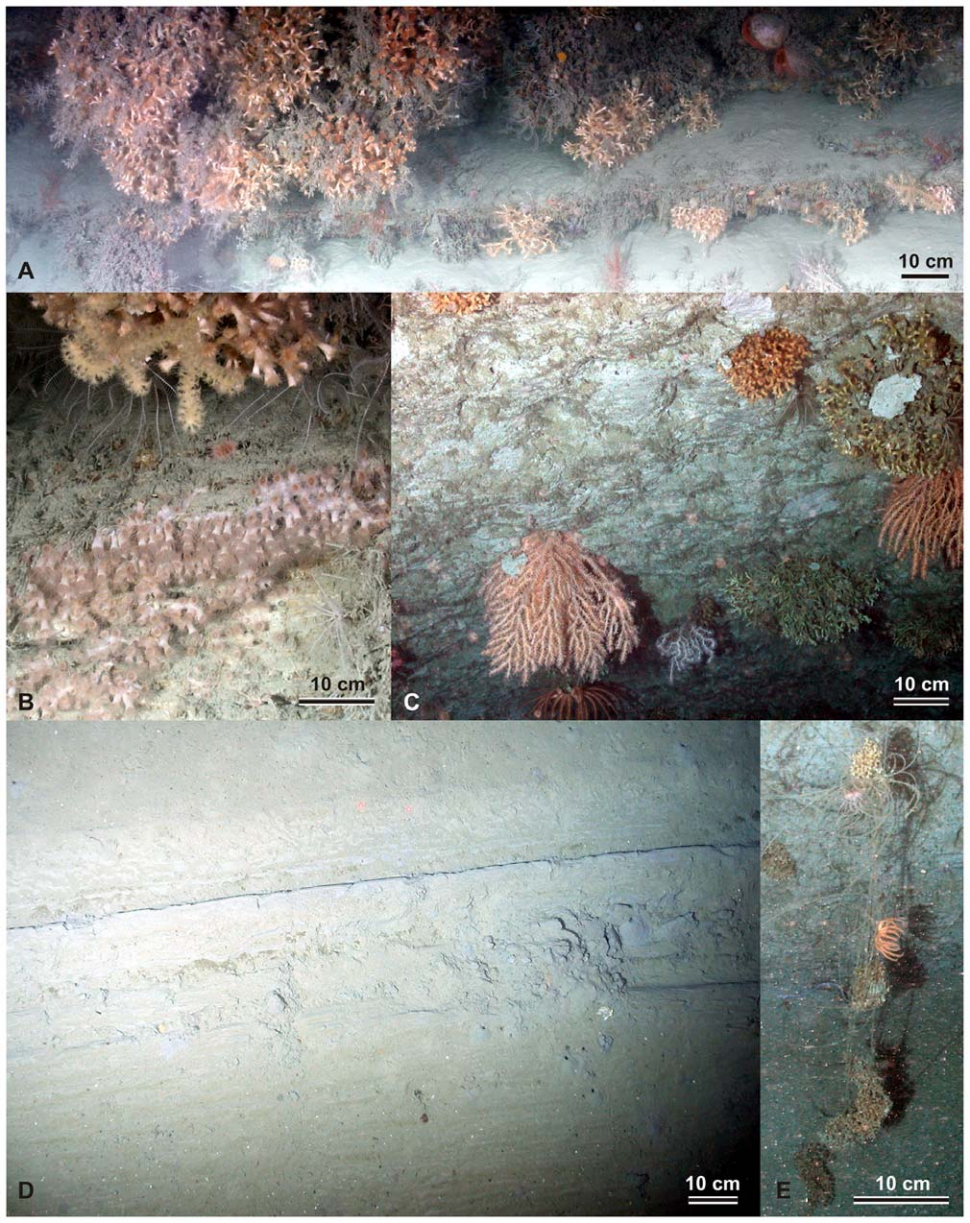
Representative seabed photographs from Whittard Canyon. **A** Large colonies of *Lophelia pertusa* at the main coral wall site D13 (1350 mwd), plus associated fauna including the large bivalve *Acesta*, several species of gorgonians, crinoids and hydroids. The fauna preferentially colonise the more resistant beds. **B** Detail of *Caryophylla* sp. at D13. **C** Coral growth on a vertical cliff in the western canyon branch, at site D02, ca. 1600 mwd. The main species are *Primnoa* sp. (large light pink colony, lower left) and *Lophelia pertusa* (small dark pink colony and larger brown colony, upper right). The substratum most probably consists of Cretaceous or Paleocene chalk, which is softer than the substratum at D13, and prone to burrowing and flaking. Note flakes of eroded rock on top of two separate coral colonies. **D** Trawl marks at ca. 550 mwd at dive site D01. **E** A Basket star (*Brisingid* sp.) and corals entangled in a lost longline at dive site D02.

The second major difference compared to the more common reef and mound habitats is that the latter build up over time through accumulation of coral rubble and baffling of sediment. Coral rubble and dead frameworks are particularly important as settling ground for new coral colonies and can host a more diverse or denser fauna than the live coral communities [6]. Coral growth on near-vertical cliffs largely excludes rubble accumulation and sediment baffling. Little rubble was observed at the foot of the cliff, suggesting it may be washed away by periodic canyon flushing events or buried by sediment. For the cliff community, the characteristics of the rocky substrata are more important. The corals preferentially settle on the more resistant beds (Fig. 4a,b), which provide a more reliable anchor point and create protruding ledges under which there is some protection against excessive sedimentation. They also allow the corals to protrude further into the flow, increasing the food encounter rate in a similar fashion to preferential coral growth on ridges and elevated substrata in horizontal settings. A comparable occurrence of *Lophelia* growth on the underside of individual outcropping hardground ledges has been reported from the NW Porcupine Bank [32]. A second site of near-vertical coral growth, although not that extensive, was found in the western branch of the Whittard Canyon, at 1515–1690 mwd (D02, Fig. 1). Here, the stepwise cliff is ca. 250 m high, and consists of relatively soft rock prone to burrowing and erosion by flaking (Fig. 4c). The coral community is dominated by *Primnoa* sp. and smaller colonies of *L. pertusa*. The reduction in colony size and density is probably because colonies break off the weak substratum when exceeding a critical size and weight.

**Figure 5:**
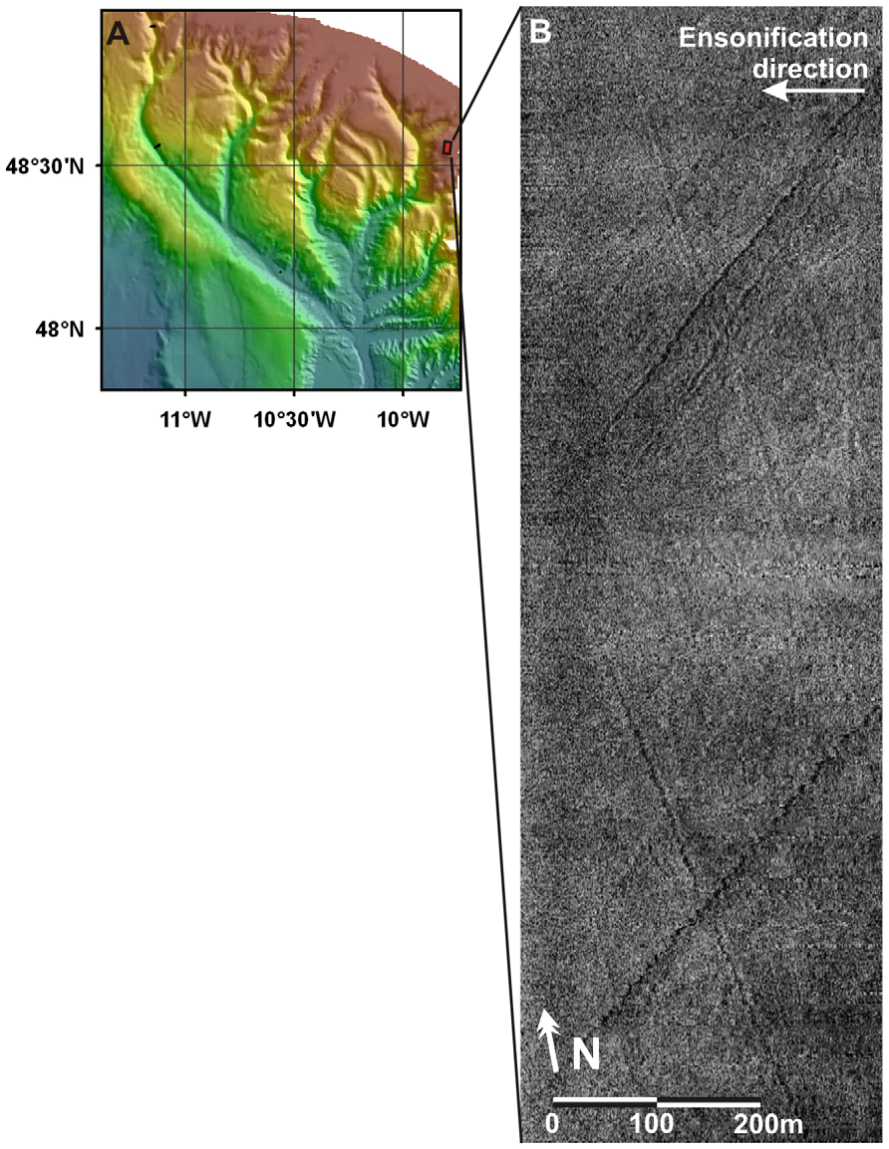
High-resolution sidescan sonar image of trawl marks on the interfluves of Whittard Canyon. **A** Location map. **B** 120 kHz sidescan sonar track illustrating a large number of criss-crossing trawl marks on the interfluves of Whittard Canyon, at ca. 250 mwd. High backscatter is represented by light colours, low backscatter by dark tones.

The nature of the vertical cliffs and overhangs means they form a natural protection against deep-sea trawling. The Bay of Biscay is a prime fishing area, and the upper continental slope is a target for several fisheries [12]. Evidence for trawl-induced seabed disturbance was observed on ROV video data (Fig. 4d) and on high-resolution sidescan sonar data (Fig. 5). However, the coral communities on the overhanging cliffs are in good condition, despite the presence of some lost long-line fishing gear (Fig. 4e). Visual inspection showed that the colonies have reached a sufficient size and maturity to support sexual reproduction [9], indicating that the vertical coral habitats could act as larval sources for the (re)colonisation of other areas along the NE Atlantic margin. Further research is necessary to establish the genetic connectivity of the Whittard corals with other populations along the margin, and to identify potential transport pathways for larval dispersal. However, it is clear that, thanks to their complex morphology, deep-sea canyons can act as refuge against anthropogenic impacts, for CWCs, and by extension for any sessile habitat-forming communities (e.g. deep-sea oysters [33]).

## Materials and Methods

This study is based on data collected in June/July 2007 and 2009, during scientific cruises JC010, JC035 and JC036 on board the RRS *James Cook*. All necessary permits were obtained for the described field studies, more specifically Diplomatic Clearance was provided by the Irish Department of Foreign Affairs to these UK-led cruises for work in the Irish sector of the Whittard Canyon.

We used a Simrad EM120 hull-mounted multibeam echosounder, the National Oceanography Centre's deeptowed 30 kHz sidescan sonar (‘TOBI’), a high-resolution EdgeTech 4200-FS dual frequency sidescan sonar (120/410 kHz), the National Environment Research Council's ROV ‘ISIS’ and a Seabird SBE9 CTD with WetLab CStar transmissometer to create a nested dataset for habitat mapping with increasing resolution for successive data layers. Shipboard multibeam data were processed with CARIS HIPS & SIPS (50 m pixel size), sidescan sonar data with the in-house developed PRISM package (3 m pixel size), and the CTD casts with the SBE software (2 m depth bins) and Ocean Data View. The ROV was equipped with 3 video cameras (including parallel lasers for scale), a digital stills camera and a Simrad SM2000 high-resolution multibeam system (200 kHz). Processing of the latter data was carried out with the IFREMER package CARAIBES (1.5 m pixel size). Video data were viewed with Final Cut Pro and classified for different coral species. All data were combined and spatial analyses were carried out in a Geographical Information System (GIS – ArcGIS software).

To map the cliff morphology, the SM2000 multibeam system of the ROV was moved from its downward-looking position to a forward-looking configuration at the front of the vehicle (Fig. S1). The cliff morphology was then mapped, navigating the ROV in lateral passes parallel to the wall. The process was repeated at different distances (60, 30, 15 and 7 m), resulting in maps with different resolutions and extent. After a double coordinate transformation of the navigation and attribute data, the multibeam swaths were processed in CARAIBES. Unfortunately, the presence of lost long-line fishing gear created an operational hazard for the ROV and video groundtruthing of the cliff had to be offset from the multibeam mapping. The scientific party decided to refrain from sampling of cliff fauna and geology in order to avoid damage to the pristine ecosystem. To estimate the percentage coral cover, a top-hat transformation [34] of the highest-resolution map was carried out to delineate background pixels from coral colonies (Fig. 2c).

Suspended particles were collected as close to the seabed as possible (within 10 m) with a stand-alone pump (SAP) on 293 mm diameter pre-combusted (400°C; 12 h) glassfibre filters. Each SAP carried two stacked filters, the bottom one being used as a dissolved organic matter (DOM) adsorption blank. On recovery, both filters were folded, wrapped in separate pre-combusted (400°C; 12 h) foil and stored at −80°C for the duration of the cruise. Sediment pushcores (6 cm diameter) were collected using the ISIS ROV, sliced in 0.5 cm (0–2 cm depth) and 1 cm (2–10 cm depth) sections and frozen at −80°C. Elemental analyses of freeze-dried SAP filters and sediments were carried out in duplicate (CEInstruments NC 2500 CHN analyser) according to the method used by Kiriakoulakis et al. [29]. Lipid analyses were carried out on freeze-dried sediment (2–3 g) or filter material (1/8 portion of the filter), which were spiked with an internal standard (5α(H)-cholestane) and extracted by sonication (×3; 30 min) with 5 mL dichloromethane: methanol (9∶1). The extracts were transmethylated with methanolic acetyl chloride solution (1 mL MeOH: AcCl; 30∶1 v/v; 45°C; overnight). GC–MS analyses were carried out on the derivatised (bis-trimethylsilyltrifluoroacetamide; 1% trimethylsilyl chloride; 50 µL; 55°C; 45 min) samples using a Trace 2000 Series gas chromatograph (on-column injector; fused silica column, 60 m×0.25 mm i.d.; 5% phenyl/95% methyl polysiloxane equivalent phase; J&W DB5-MS or ZB5-MS; carrier gas helium at 1.6 mL min^−1^), coupled with a Thermoquest Finnigan TSQ7000 mass spectrometer (ionisation potential 70 eV; source temperature 215°C; trap current 300 µA) and processed using Xcalibur software. Compounds were identified by comparison of their mass spectra and relative retention indices with those available in the literature and/or by comparison with authentic standards. Quantitative data were calculated by comparison of peak area of the internal standard with those of the compounds of interest, using the total ion current (TIC) chromatogram. The relative response factors of the analytes were determined individually for 36 representative fatty acids, sterols and alkenones using authentic standards. Response factors for analytes where standards were unavailable were assumed to be identical to those of available compounds of the same class. Reproducibility of the lipid extraction procedure and analysis are reported by Jeffreys et al. [35].

## Supporting Information

Figure S1 Photograph of ISIS ROV with the SM2000 receiving transducer mounted on the front of the vehicle.
(TIF)
